# The CRUMB study: closed-loop response to unannounced mixed and carbohydrates-rich breakfasts. A randomized controlled crossover pilot-study in a cohort of adolescents with type 1 diabetes

**DOI:** 10.1007/s12020-026-04705-5

**Published:** 2026-07-10

**Authors:** Donatella Lo Presti, Salvatore Scirè Calabrisotto, Letizia Grazia Tomaselli, Giulia Pezzino, Francesco Frasca, Roberto Baratta, Laura Sciacca, Concetta Latina, Nadia La Spina, Francesco Galeano, Vittorio Oteri, Andrea Tumminia

**Affiliations:** 1 Regional Reference Center of Paediatric Diabetology, Policlinico “G. Rodolico” Hospital, Catania, Italy; 2 Paediatric Medicine Section, Department of Clinical and Experimental Medicine, University of Catania, Catania, Italy; 3 Paediatric Diabetology Unit, Maternal and Child Health Department, Garibaldi- Nesima Hospital, Catania, Italy; 4 Endocrinology Section, Department of Clinical and Experimental Medicine, University of Catania, Garibaldi-Nesima Hospital, Catania, Italy; 5 Endocrine Unit, Garibaldi-Nesima Hospital, Catania, Italy

**Keywords:** Type 1 diabetes, Automated insulin delivery, Adolescents, Unannounced meals, Proportional integral derivative controller, Model predictive control

## Abstract

**Purpose:**

Unannounced meals pose a major challenge to type 1 diabetes patients. This study compared the performance of two automated insulin delivery (AID) algorithms, the Medtronic MiniMed 780G’s proportional-integral-derivative (PID) and the Tandem t: slim X2’s model predictive control (MPC), following unannounced breakfasts (ClinicalTrials.gov identifier: NCT07455643, retrospectively registered the 3rd of February 2026).

**Methods:**

In a randomized crossover study, we enrolled 20 children between 11 and 18 years using the MiniMed 780G or the Tandem t: slim X2 AID system. Endpoints included 2-hour and 4-hour blood glucose difference (ΔBG), glucose peak, time to peak, and time spent above (TAR), below (TBS), in range (TIR), and in tight range (TITR). Two meals were tested: a carbohydrate (CHO) meal and a mixed one, both containing 30 g of carbohydrates, and additional 15 g proteins for the mixed meal. Announced (AM) and unannounced (UM) meals were analyzed.

**Results:**

AM, compared to UM, showed significant higher 2-hourΔBG; conversely 4-hourΔBG did not differ significantly. In CHO AM, algorithms were comparable. In mixed AM, PID gained lower peaks and TAR, with higher TIR and TITR. In UM, PID obtained lower 2-hourΔBG than MPC, with reduced peak, TAR, and improved TIR and TITR. Differences in 4-hourΔBG and time-at-peak were not significant.

**Conclusion:**

Both AID algorithms mitigated postprandial glycemia and returned glucose to baseline levels within 4 h without safety concerns. The PID demonstrated higher reactivity to unannounced meals, while performances were comparable when meal boluses were properly announced.

**ClinicalTrials.gov identifier:**

NCT07455643.

**Supplementary Information:**

The online version contains supplementary material available at 10.1007/s12020-026-04705-5.

## Introduction

Automated insulin delivery (AID) systems represent the actual gold standard in type 1 diabetes (T1D) management. These systems, through the algorithm integrated in the insulin pumps, utilize continuous glucose monitoring (CGM) device readings to automatically adjust insulin delivery, therefore reducing both hypo-and hyperglycemic events and improving overall glycemic control [1, 2]. AID use has also been linked to a reduced diabetes-related burden in people with diabetes (PwD) and their caregiver, adding to the better glucometabolic control an enhanced perceived quality of life [3]. However, despite these technological advances, a relevant fraction of T1D patients do not achieve the recommended glycemic goals [4, 5], suggesting that everyday challenges faced by PwD are not completely managed by AID systems.

Therapeutic adherence is still a core and burdensome factor in T1D. Daily self-management requires meticulous attention to carbohydrate counting, timely meal bolusing, and adjustment for physical activity or stress, all of which can be demanding for PwD and their families [6, 7]. Errors in carbohydrate estimation or missed boluses are among the most common issues encountered in clinical practice [8–10]. Such episodes may be a consequence of simple forgetfulness, or, additionally, they could also depend on complex psychosocial factors related to diabetes-induced burnout, cognitive overload, or linked to the social and self-stigma of insulin administration [9–12]. This leads to suboptimal glucose control [13, 14], and, overall, a more relevant long-term complication risk.

The development of advanced hybrid closed-loop (a-HCL) systems represents a significant step toward in improving glucose control and reducing user-dependent variability, especially in pediatric patients [15–17]. A-These systems can automatically deliver correction boluses and modulate insulin delivery based on CGM feedback, thereby compensating for some of the consequences of human error. Up to date evidence suggests that a-HCL systems can tolerate unannounced carbohydrate loads up to approximately 20 g without compromising time in range (TIR) or safety [18–20]. However, the metabolic response to larger or compositionally complex meals remains variable and highly dependent on the specific algorithm governing insulin delivery.

Currently a variety of AID are commercially available: all of them present similarities and differences. The Medtronic MiniMed 780G uses a proportional–integral–derivative (PID) algorithm, a mathematical model that adjusts in real time insulin delivery rate based on 3 elements obtained from CGM reading values: the difference between the actual value and the chosen glucose target (proportional action), past values (integral action) and the glucose’s rate of change (derivative action). In contrast, the Tandem t: slim X2 with Control-IQ employs a model predictive control (MPC) algorithm, which aims, through a complex mathematical model, to predict glucose trends up to half-an-hour in the future, taking, also, in consideration actual and past glucose values. Despite sharing the same objective, said algorithms have different approaches, the former one being “reactive” and the latter “predictive”. Therefore, their difference could result in different performances while facing mixed-nutrient meal or unannounced meals, defined as the consumptions of a meal with any prior insulin administration.

Pediatric patients represent certainly a unique subgroup in which therapeutic adherence is a relevant issue, due to cognitive, developmental and behavioral factors. Understanding how different AID algorithms respond to unannounced meals in this age group is therefore crucial to ensure safety and personalization of diabetes management.

This study was designed to evaluate the strengths and limitations of two a-HCL systems, the Medtronic 780G (PID algorithm) and the Tandem t: slim X2 (MPC algorithm), in managing unannounced meals with different macronutrient compositions in children and adolescents with T1D. We also aim to better understand physiological and technological unannounced meal implications as to provide additional insight useful for the development of new fully closed loop algorithms, capable of minimizing glucose excursions and patient’s burden.

## Methods

### Study design and protocol

This investigation was designed as a two-arm, randomized, open label, crossover, controlled pilot study. We followed the Consolidated Standards of Reporting Trials (CONSORT) Statement [21] for reporting of our trial (Appendix 1 in the Online Resource).

Participants were recruited from two paediatric diabetes centers in Catania, Italy: the Paediatric Diabetes Regional Reference Centre of the A.O.U. Policlinico “G. Rodolico – S. Marco” and the Paediatric Diabetology Unit of the A.R.N.A.S. “Garibaldi - Nesima” Hospital.

All study procedures were conducted according to the 1964 Declaration of Helsinki and subsequent amendments. The study protocol was formally submitted to the National Pediatric Ethics Committee for regulatory oversight and approval. Then, to ensure maximal transparency and public accountability, the trial was registered at ClinicalTrials.gov (Identifier: NCT07455643, https://clinicaltrials.gov/study/NCT07455643, retrospectively registered the 3rd of February 2026), adhering to international standards for clinical research reporting.

### Participants selection

We enrolled adolescents with T1D with an age range between 11 and 18 years old. All included participants were treated with one of the two a-HCL system, the Medtronic MiniMed 780G or the Tandem t: slim X2. Other mandatory inclusion criteria were the following: demonstrated competence in carbohydrates counting, at least 3 months of a-HCL device utilization, and a T1D diagnosis confirmed at least 1 year before study procedures. Conversely patients with history of malabsorptive disease or diabetes-relates complications were excluded, as to control confounding factors derived by altered nutrient absorption or glucometabolic settings.

### Study procedures

Families were initially contacted and informed about the objectives and phases of the study. Those who agreed to participate provided written informed consent prior to their inclusion in the study and attended a subsequent meeting in which detailed instructions were given regarding study procedures, device management, and the meal testing protocol.

Randomization was generated by a central computer, and stratified by sex, device type, and recruiting center to ensure balanced group distribution. Participants were then assigned to two groups: Group A first underwent the announced meal (AM) condition followed, after a seven-day washout period, by the unannounced meal (UM) condition; Group B followed the opposite order.

All participants were required to consume two standard breakfast meals selected from our center’s dieticians. The first one, referred to as the carbohydrate-based (CHO) meal, consisted of 40 g of white bread (23,80 g CHO, 0,20 g fats, 3,25 g proteins) together with 10 g jam (6,95 g CHO) summing up to 30,75 g CHO. The second meal, referred as “Mixed meal”, consisted of 50 g of white bread with 40 g of Italian bresaola (15,2 g proteins, 0,8 g fats), for a total of 30 g CHO and 15 g of proteins. Both study meals were chosen intentionally low-in-fats meals to minimize lipid impact on the post-prandial blood glucose curve. Nutritional values were obtained from the CREA (Council for Agricultural Research and Economics) food composition database [22].

**Fig. 1:**
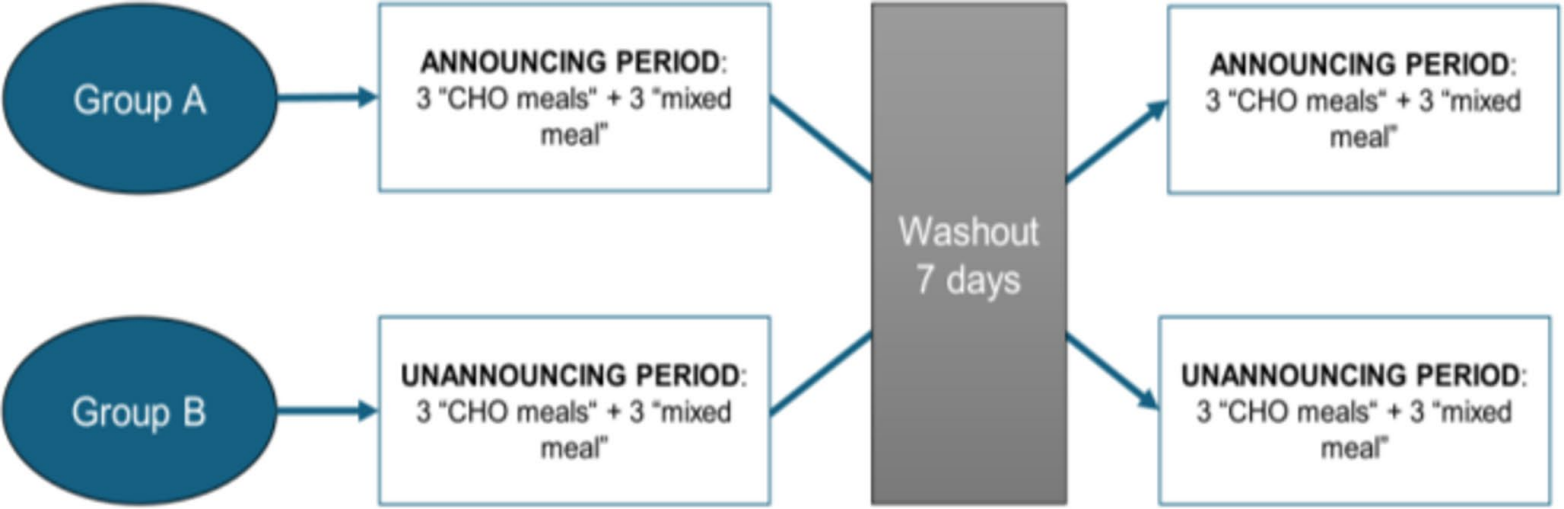
Study design

In an at-home setting, each participant completed three distinct study phases. During the first phase, referred to as the announcing period, participants followed their usual therapy while consuming both the “CHO meal” and the “mixed meal” three times each. In this phase, the carbohydrate content of each meal was announced to the insulin pump, and pre-meal boluses were delivered as per standard practice. The second phase, termed the unannouncing period, required participants to consume the same meals, an equal number of times, without announcing their carbohydrate intake to the device. Consequently, no user-initiated boluses were administered during this period, and all insulin delivery adjustments were determined exclusively by the algorithm. A seven-day washout interval separated the two phases. Overall, each participant consumed a total of twelve study meals. The only planned difference between the two randomized groups concerned the order of these phases: Group A began with the announcing period and then proceeded to the unannouncing period, while Group B followed the opposite sequence. Figure 1 depicts the study design.

Throughout the entire study, participants were instructed not to interact with or modify the insulin pump settings for at least three hours before and four hours after each test meal. This ensured that insulin delivery during the observation window reflected the system’s automated algorithmic response, except for the manual bolus given during the announcing phase. Participants were instructed to withhold study procedures if pre-meal glucose value were below 80 mg/dl or above 150 mg/dl and to resume the following day. The same protocol has been advised if patients showed fever, or any acute illness capable of affecting glucose metabolism or if hypoglycemia had occurred within the two hours preceding the study meal. As safety procedures, patients were encouraged to intervene in the case of CGM values over 300 mg/dl, or below 70 mg/dl in the presence of hypoglycemia symptoms. To minimize confounding effects related to prior food intake or daily activity, all study meals were consumed at breakfast. At the completion of the third study phase, data were downloaded and analyzed.

### Outcomes

The primary study outcome was the change in blood glucose concentration from pre-meal to two hours post-meal (2 h-ΔBG), as measured by CGM. Values were obtained from Dexcom G6 CGM sensor, for participants using MPC algorithm, and the Guardian 4 sensor, for patients using PID algorithm. Pre- and post-meal four-hour glucose difference (4 h-ΔBG), as well as peak CGM value, time to peak, and percentages of spent time in range (TIR, 70–180 mg/dL), time in tight range (TITR, 70–140 mg/dL), time below range (TBR, < 70 mg/dL), time above range (TAR, > 180 mg/dL), and second-level TAR (> 250 mg/dL) were assessed as secondary outcomes and evaluated in the four hour postprandial.

### Statistical analysis

Sample size estimation has been calculated on the primary endpoint, 2 h-ΔBG, between announced and unannounced meals. Preliminary data indicated a paired mean difference of approximately 40 mg/dL and a standard deviation of 45 mg/dL. Assuming 80% power, with two-tailed α = 0.05, and a within-subject correlation of 0.4, we estimated 12 participants as a required sample size. To minimize the impact of potential dropouts or missing data, a total of 20 subjects were enrolled, ensuring adequate statistical power.

Relevant data were exported .csv files from the Glooko and CareLink online platforms. In order to verify adherence to the study procedures, reports were reviewed. Participants were asked to repeat the test meal, in case of discrepancies from the given instructions. The raw CGM data files were then processed to obtain 2 h-ΔBG, 4 h-ΔBG, peak glucose, time to peak, TIR, TBR, TAR, > 250 TAR, and TITR for all twelve study-meal. These values were consolidated into a dedicated database for subsequent statistical processing.

Subsequently, comparative analyses were executed for all outcomes in all relevant cases, namely: announced versus unannounced meals (AM vs. UM), CHO AM versus CHO UM, mixed AM versus mixed UM, CHO AM versus mixed AM, and CHO UM versus mixed UM. All comparisons performed for the total study cohort was further repeated for the MPC and PID subgroups. Paired-sample t-tests were used for within-subject analyses.

Further statistical analyses were conducted comparing all outcomes between the PID and MPC subgroups, and between Group A and Group B. To compare PID and MPC algorithms Welch’s t-test was applied, whereas Student’s t-test was used to compare Group A and Group B. Data was processed using Microsoft Excel (Microsoft Corp.) and STATA (StataCorp LLC) software. Results are reported as mean ± standard deviation, considering *p* < 0.05 as statistically significant.

## Results

Initially, 40 patients were contacted, and 20 of them agreed to participate in the study procedures. A total of 20 participants successfully completed the full study protocol and were further included for the final analysis. The study duration was one month, from the 20th of April 2025 to the 20th of May 2025.

**Fig. 2:**
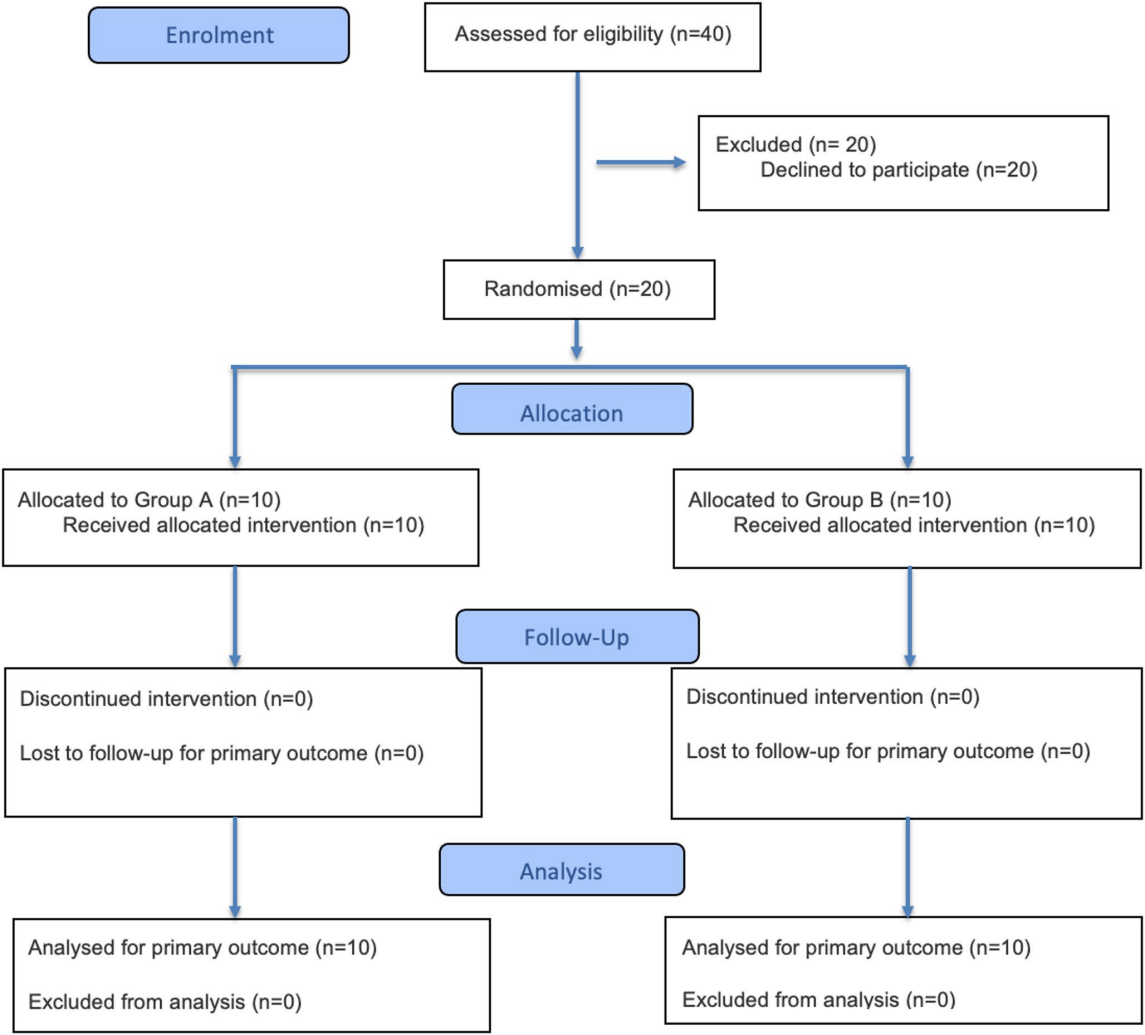
CONSORT 2025 flow diagram

No serious adverse events occurred during the study, including diabetic ketoacidosis or severe hypoglycemia. Figure 2 depicts the CONSORT 2025 flow diagram.

Table 1 depicts the baseline characteristics of the study population.

**Table 1:** Baseline population characteristics for Group A and B

	Group A		Group B		
	Mean	SD	Mean	SD	*p*-value for t-test
Baseline HbA1c	6,8	0,6	6,9	0,3	0,714
TIR	78,4	6,1	73,7	5,9	0,097
TAR	19,1	6,6	24,5	6,2	0,075
TBR	2,5	2,0	1,8	1,1	0,341
CV	32,9	4,7	34,4	4,1	0,445
Age	14,5	1,8	14,6	1,8	0,905
Weight	63,1	13,0	56,3	7,9	0,175
Height	1,7	0,1	1,6	0,1	0,491
BMI	22,3	3,2	20,9	2,7	0,306
	**n**		**n**		
Total	10		10		
Male	7		6		
Female	3		4		
PID	5		5		
MPC	5		5		
CRR-Policlinico	7		6		
UOSD-Nesima	3		4		

### Primary outcome

**Fig. 3:**
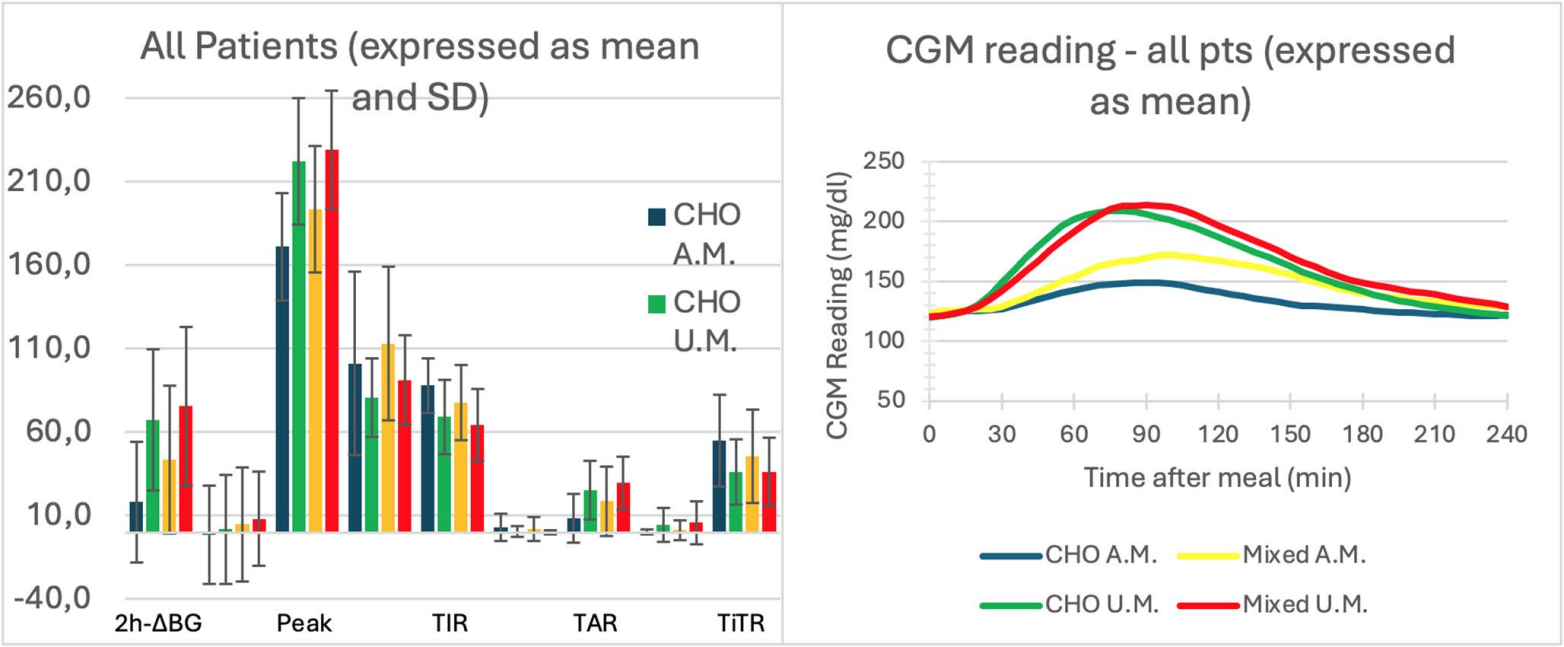
All patients population sample results. **A** Mean CGM readings for each meal. **B** Column chart of outcomes results representing mean and standard deviation. Abbreviations: all pts, all patients. A.M., announced meals. U.M., unannounced meals. 2 h-ΔBG, 2-hour delta blood glucose. 4 h-ΔBG, 4-hour delta blood glucose. T. Peak, time at peak. TIR, time in range. TBR, time below range. TAR, time above range. TiTR, time in tight range

AM and UM comparison of 2 h-ΔBG across the total sample showed a highly significant difference (30.9 ± 42.3 vs. 71.5 ± 45.0 mg/dL, *p* < 0.001). These patterns were consistent for both MPC and PID subgroups, showing a higher postprandial rise during UM and mixed meals, comparing them with AM and CHO meals. CHO UM and mixed UM comparison did not achieve statistical significance in any group (all patients: 67.3 ± 42.2 vs. 75.6 ± 47.6 mg/dL, *p* = 0.205) (Table 2; Fig. 3; Table 1s, 2s, and 3s and Figs. 2s, and 3s of the Online Resource).

**Table 2:** All patients sample population statistical analysis

	All pts A.M. vs. U.M.	All pts CHO A.M. vs. U.M.	All pts Mixed A.M. vs. U.M.	All pts CHO A.M. vs. Mixed A.M.	All pts CHO U.M. vs. Mixed U.M.
**2h-ΔBG**	30,9 ± 42,3 vs. 71,5 ± 45,0 (*p* < 0,001)	18,2 ± 36,3 vs. 67,3 ± 42,2 (*p* < 0,001)	43,7 ± 44,3 vs. 75,6 ± 47,6 (*p* < 0,001)	18,2 ± 36,3 vs. 43,7 ± 44,3 (*p* < 0,001)	67,3 ± 42,2 vs. 75,6 ± 47,6 (*p* = 0,205)
**4h-ΔBG**	1,8 ± 31,9 vs. 5,0 ± 30,5 (*p* = 0,384)	-1,3 ± 29,4 vs. 1,9 ± 32,6 (*p* = 0,540)	4,8 ± 34,2 vs. 8,1 ± 28,3 (*p* = 0,541)	-1,3 ± 29,4 vs. 4,8 ± 34,2 (*p* = 0,231)	1,9 ± 32,6 vs. 8,1 ± 28,3 (*p* = 0,235)
**Peak**	182,4 ± 36,7 vs. 225,6 ± 36,5 (*p* < 0,001)	171,2 ± 32,2 vs. 222,2 ± 37,7 (*p* < 0,001)	193,6 ± 37,7 vs. 229,1 ± 35,3 (*p* < 0,001)	171,2 ± 32,2 vs. 193,6 ± 37,7 (*p* < 0,001)	222,2 ± 37,7 vs. 229,1 ± 35,3 (*p* = 0,187)
**T.Peak**	107,0 ± 50,8 vs. 85,9 ± 25,6 (*p* < 0,001)	101,2 ± 54,9 vs. 80,6 ± 23,5 (*p* = 0,009)	112,9 ± 46,0 vs. 91,2 ± 26,7 (*p* = 0,001)	101,2 ± 54,9 vs. 112,9 ± 46,0 (*p* = 0,209)	80,6 ± 23,5 vs. 91,2 ± 26,7 (*p* = 0,023)
**TIR**	82,9 ± 20,2 vs. 66,8 ± 21,9 (*p* < 0,001)	88,0 ± 16,4 vs. 69,2 ± 22,4 (*p* < 0,001)	77,8 ± 22,3 vs. 64,3 ± 21,4 (*p* < 0,001)	88,0 ± 16,4 vs. 77,8 ± 22,3 (*p* = 0,002)	69,2 ± 22,4 vs. 64,3 ± 21,4 (*p* = 0,138)
**TBR**	2,5 ± 7,8 vs. 0,4 ± 2,5 (*p* = 0,005)	3,0 ± 8,3 vs. 0,7 ± 3,2 (*p* = 0,039)	2,0 ± 7,2 vs. 0,2 ± 1,3 (*p* = 0,060)	3,0 ± 8,3 vs. 2,0 ± 7,2 (*p* = 0,360)	0,7 ± 3,2 vs. 0,2 ± 1,3 (*p* = 0,167)
**TAR**	13,7 ± 18,6 vs. 27,6 ± 16,8 (*p* < 0,001)	8,6 ± 14,7 vs. 25,4 ± 17,6 (*p* < 0,001)	18,8 ± 20,8 vs. 29,7 ± 15,9 (*p* = 0,001)	8,6 ± 14,7 vs. 18,8 ± 20,8 (*p* = 0,001)	25,4 ± 17,6 vs. 29,7 ± 15,9 (*p* = 0,174)
**> 250 TAR**	0,8 ± 4,5 vs. 5,1 ± 11,5 (*p* < 0,001)	0,3 ± 1,7 vs. 4,5 ± 10,2 (*p* = 0,003)	1,3 ± 6,1 vs. 5,7 ± 12,7 (*p* = 0,009)	0,3 ± 1,7 vs. 1,3 ± 6,1 (*p* = 0,236)	4,5 ± 10,2 vs. 5,7 ± 12,7 (*p* = 0,473)
**TITR**	50,4 ± 28,0 vs. 36,2 ± 19,9 (*p* < 0,001)	55,1 ± 27,4 vs. 36,1 ± 19,7 (*p* < 0,001)	45,7 ± 27,9 vs. 36,3 ± 20,3 (*p* = 0,009)	55,1 ± 27,4 vs. 45,7 ± 27,9 (*p* = 0,019)	36,1 ± 19,7 vs. 36,3 ± 20,3 (*p* = 0,970)

Abbreviations: *all pts* all patients, *A*.*M*. announced meals, *U*.*M*. unannounced meals, *2h*-Δ*BG* 2-hour delta blood glucose, *4h*-Δ*BG* 4-hour delta blood glucose, *T*. *Peak* time at peak, *TIR* time in range, *TBR* time below range, *TAR* time above range, *TiTR* time in tight range

During algorithm devices comparisons, PID returned a significantly smaller 2 h-ΔBG values in contrast to the MPC algorithm for all meals (43.5 ± 38.4 vs. 58.9 ± 55.1 mg/dL, *p* = 0.013), for UM (55.6 ± 36.4 vs. 87.3 ± 47.4 mg/dL, p < 0.001), for CHO UM (51.1 ± 36.4 vs. 83.5 ± 41.8 mg/dL, *p* = 0.002), and for the mixed UM (60.1 ± 36.4 vs. 91.1 ± 52.7 mg/dL, *p* = 0.011). Other comparisons evaluated between algorithms were found not statistically significant (Table 1s and Fig. 1s of Online Resource).

Group A versus group B comparison yielded a significant difference in 2 h-ΔBG in the following cases: all patients (45.1 ± 4.8 vs. 57.3 ± 50.3 mg/dL, *p* = 0.047), UM (63.1 ± 39.3 vs. 79.8 ± 49.9 mg/dL, *p* = 0.042), and mixed UM (60.0 ± 36.0 vs. 91.2 ± 53.0 mg/dL, *p* = 0.010). Other comparisons, between groups A and B (AM, CHO AM, CHO UM, or mixed AM) reached statistical significance (Table 4s and Fig. 4s of Online Resource).

### Secondary outcomes

Secondary endpoints included 4 h-ΔBG, peak glucose, time to peak, TIR, TBR, TAR, > 250 TAR, and TiTR. (See Table 2, and Fig. 1; Table 1s, 2s, 3s, and 4s, and Figs. 1s, 2s, 3s and 4s in the Online Resource).

No significant differences in 4 h-ΔBG were observed across comparisons or populations (e.g., all patients AM vs. UM: 1.8 ± 31.9 vs. 5.0 ± 30.5 mg/dL, *p* = 0.384; PID vs. MPC: 3.0 ± 26.1 vs. 3.7 ± 35.7 mg/dL, *p* = 0.863). Only group A vs. group B comparison involving AM showed significance.

Across the total population, peak glucose values were significantly higher during UM and mixed meals than during AM and CHO meals, respectively, except for CHO UM vs. mixed UM (222.2 ± 37.7 vs. 229.1 ± 35.3 mg/dL, *p* = 0.187). A similar trend was found in the MPC group, while in the PID group, CHO AM vs. mixed AM and CHO UM vs. mixed UM comparisons were not significant. Peak levels were generally lower in PID users compared with MPC users, except during AM and CHO AM, where no significant differences emerged.

In all patients, MPC, and PID populations analysis, UM and CHO meals showed earlier peaks when confronted with AM and mixed meals (e.g., all patients AM vs. UM: 107.0 ± 50.8 vs. 85.9 ± 25.6 min, p < 0.001; CHO UM vs. mixed UM: 80.6 ± 23.5 vs. 91.2 ± 26.7 min, *p* = 0.023). without any significant differences found between PID vs. MPC or A vs. B comparisons.

AM returned significantly higher TIR percentages across all population’s samples (all patients AM vs. UM: 82.9 ± 20.2 vs. 66.8 ± 21.9%, p < 0.001). PID algorithm, generally, achieved higher TIR than MPC in nearly all comparisons, exception made for CHO AM (89.7 ± 16.1 vs. 86.4 ± 16.9%, *p* = 0.446).

AM and CHO AM, across all patient population sample, showed higher TBR relative to, respectively, UM and CHO UM. Only MPC AM vs. UM reached significance (4.2 ± 10.3 vs. 0.5 ± 2.8%, *p* = 0.007). In PID vs. MPC comparisons, differences were found significant for all meals (0.6 ± 2.7 vs. 2.3 ± 7.7%, *p* = 0.021) and AM (0.8 ± 3.2 vs. 4.2 ± 10.3%, *p* = 0.017).

TAR percentages were consistently higher in UM compared with AM (all patients: 13.7 ± 18.6 vs. 27.6 ± 16.8%, p < 0.001). MPC users showed higher TAR than PID users in most comparisons, though only a subset reached significance. TAR > 250 mg/dL was significantly greater in UM relative to AM in most analyses (all patients AM vs. UM: 0.8 ± 4.5 vs. 51.1 ± 11.5%, p < 0.001). No significant differences emerged in PID users. PID and MPC algorithms showed similar control during AM, whereas UM results favored the PID algorithm.

As expected, TITR was lower for UM and mixed meals compared to AM and CHO meals (all patients: 50.4 ± 28.0 vs. 36.2 ± 19.9%, p < 0.001). This pattern held across populations. During AM, both algorithms performed similarly, while during UM, the PID algorithm consistently achieved higher TITR, except for mixed UM, where no significant difference was observed

## Discussion

As expected, unannounced meals adversely affected postprandial glucose profiles, leading to higher 2 h-ΔBG values, elevated and earlier glucose peaks, and overall deterioration in glycemic metrics (TIR, TAR, > 250 TAR, and TiTR), with the exception of TBR. Nonetheless, a reassuring finding was that both algorithms were consistently able to restore glycemia to pre-prandial levels within four hours. This suggests that, for meals containing approximately 30 g of carbohydrates, the short-term safety of individuals using a-HCL systems is not compromised even in the absence of meal announcement. However, repeated omissions of boluses over time could still have deleterious effects on long-term glycemic control and complication risk.

No significant differences were observed between unannounced carbohydrate-rich and mixed carbohydrate-protein meals for any of the evaluated outcomes, suggesting that within this modest nutrient range, both algorithms manage unannounced intake with comparable efficiency. The only significant difference emerged in the time to glucose peak, reflecting the physiological delay introduced by protein ingestion—an observation consistent with previous findings [23]. However, while correctly administrating an announced bolus, both algorithms performed better in carbohydrate-rich meals than mixed ones, generally, underlying macronutrient composition influence on the algorithm response.

Expected physiological differences in CHO and mixed meals were observed, as the latter produced higher but delayed glycemic peaks. Proteins impact on decelerating glucose absorption could be beneficial only if the insulin dose accounts also for protein metabolism. These findings suggest the need for simplified insulin dosing approaches that includes also protein and fat content, other than carbohydrates. Extending this research to mixed carbohydrate–fat meals would further clarify how dietary composition affects unannounced meal management.

The PID and MPC algorithms performed similarly across most conditions, particularly when meals were properly announced, though both systems showed limitations when faced with unannounced intake: unsurprising given that neither was originally designed for such situations. While both mitigated glycemic excursions and prevented dangerous deviations, they were unable to fully prevent hyperglycemia or maintain TIR at baseline levels. Nonetheless, this capacity to partially correct unannounced excursions represent a meaningful advancement toward fully closed-loop systems.

Interestingly, the PID algorithm outperformed the MPC algorithm during unannounced meals, achieving better outcomes across several metrics, including 2 h-ΔBG, glucose peak, TIR, TAR, TBR, and TiTR. This difference may be explained by the Tandem t: slim X2’s conservative correction protocol, which administers only 60% of the calculated correction bolus and limits automatic corrections to once per hour. Consequently, its response to unannounced meals or suboptimal boluses is inherently slower. In contrast, the PID algorithm used in the Medtronic Minimed 780G can deliver automated correction boluses every five minutes once the basal rate limit is reached, allowing for a faster and more reactive glycemic response. This feature may be particularly advantageous for less adherent patients, potentially reducing the risk of long-term complications. However, since the Tandem t: slim X2 can be upgraded via software updates, future improvements could narrow this performance gap at no additional cost, an important consideration in the context of public healthcare resource allocation.

Looking ahead, the management of unannounced meals remains one of the greatest challenges in the development of fully closed-loop systems. Algorithms capable of detecting missed boluses and delivering appropriate corrective insulin doses in real time should be a design priority. Carbohydrate counting, and therefore bolus calculation, is a pillar of diabetes management, which still needs frequent and system-specific reassessment [24]. Unfortunately, this is a major source of treatment burden, particularly among children and adolescents, due to the cognitive and mathematical demands involved [6, 7]. Although current a-HCL systems allow for more flexible and less precise carbohydrate estimation [25], it should be underlined that automatisms actually helps and do not substitute the user in glucose managing, as an higher correction boluses over total daily boluses, the Automated Correction Index, is correlated to worse glucose metrics [26] even if, in terms of efficiency, automated correction boluses achieve better control over manual boluses, especially when insulin dosing variability is considered [27]. Future devices should effectively manage protein and fat impact on blood glucose, thereby reducing patients’ burden while enhancing overall control. In addition, a newer ultra-rapid insulin formulation might be decisive for the management of delayed or unannounced boluses, and overall enhancing devices responsiveness.

### Strengths and limitations

This study has several strengths. To our knowledge, it is the first to compare the response to unannounced meals between two among the most widely used a-HCL systems, providing direct insight into their comparative performance. It also contributes novel information on glycemic dynamics, including ΔBG and glucose curve parameters, and offers additional safety data regarding automated correction performance.

On the other hand, limitations include our relatively small sample size, the non-controlled at-home environment, and the use of two different CGM systems. Intergroup variability might be reduced by the involvement of a larger cohort and strengthened the observed trends. Finally, it is important to acknowledge the relatively short duration of our study (one month). Whereas this timeframe provided sufficient data to compare the immediate physiological responses and algorithmic reactivity of the PID and MPC systems to unannounced meals, it may not fully capture long-term glycemic trends, potential “user fatigue” with the utilization of AID systems, or seasonal variations in insulin sensitivity. Further longitudinal studies are warranted to confirm whether the observed differences in postprandial control persist over extended periods of real-world use.

## Conclusions

Both AID algorithms mitigated postprandial glycemia and returned glucose to baseline levels within 4 h without safety concerns. The PID demonstrated higher reactivity to unannounced meals, while performances were comparable when meal boluses were properly announced.

PID and MPC both proved themselves to be safe and adaptive algorithms, being capable of safely managing small unannounced carbohydrates loads. Future developments in fully closed-loop systems should aim to enhance reactivity and precision to prevent hyperglycemia following unannounced intake. Reactiveness, when balanced with hypoglycemia prevention, appears to be a key determinant of optimal glucose control and may ultimately play a central role in reducing long-term diabetes-related complications.

## Supplementary Information

Below is the link to the electronic supplementary material.

Supplementary Material 1

## Data Availability

The datasets generated during and/or analysed during the current study are available from the corresponding author on reasonable request.
